# Shoal familiarity modulates effects of individual metabolism on vulnerability to capture by trawling

**DOI:** 10.1093/conphys/coz043

**Published:** 2019-07-26

**Authors:** J P W Hollins, D Thambithurai, T E Van Leeuwen, B Allan, B Koeck, D Bailey, S S Killen

**Affiliations:** 1 Institute of Biodiversity, Animal Health and Comparative Medicine, Graham Kerr Building, College of Medical, Veterinary and Life Sciences, University of Glasgow, Glasgow G12 8QQ, UK; 2 Fisheries and Oceans Canada, Salmonid Section, 80 East White Hills Road, PO Box 5667, St. John’s, Newfoundland A1C 5X1, Canada; 3 Department of Ocean Sciences, Memorial University of Newfoundland, St. John’s, Newfoundland A1C 5S7, Canada

**Keywords:** Ecophysiology, fishing, metabolism, social behaviour, trawl

## Abstract

Impacts of fisheries-induced evolution may extend beyond life history traits to more cryptic aspects of biology, such as behaviour and physiology. Understanding roles of physiological traits in determining individual susceptibility to capture in fishing gears and how these mechanisms change across contexts is essential to evaluate the capacity of commercial fisheries to elicit phenotypic change in exploited populations. Previous work has shown that metabolic traits related to anaerobic swimming may determine individual susceptibility to capture in trawls, with fish exhibiting higher anaerobic performance more likely to evade capture. However, high densities of fish aggregated ahead of a trawl net may exacerbate the role of social interactions in determining an individual fish’s behaviour and likelihood of capture, yet the role of social environment in modulating relationships between individual physiological traits and vulnerability to capture in trawls remains unknown. By replicating the final moments of capture in a trawl using shoals of wild minnow (*Phoxinus phoxinus*), we investigated the role of individual metabolic traits in determining susceptibility to capture among shoals of both familiar and unfamiliar conspecifics. We expected that increased shoal cohesion and conformity of behaviour in shoals of familiar fish would lessen the role of individual metabolic traits in determining susceptibility to capture. However, the opposite pattern was observed, with individual fish exhibiting high anaerobic capacity less vulnerable to capture in the trawl net, but only when tested alongside familiar conspecifics. This pattern is likely due to stronger cohesion within familiar shoals, where maintaining a minimal distance from conspecifics, and thus staying ahead of the net, becomes limited by individual anaerobic swim performance. In contrast, lower shoal cohesion and synchronicity of behaviours within unfamiliar shoals may exacerbate the role of stochastic processes in determining susceptibility to capture, disrupting relationships between individual metabolic traits and vulnerability to capture.

## Teaser

The role of shoal familiarity in modulating relationships between individual fish’s metabolic traits and vulnerability to capture in a simulated trawl were tested in the lab. Fish tested in familiar shoals showed a negative relationship between anaerobic metabolic capacity and time spent in the trawl net, but not fish tested in unfamiliar shoals.

## Introduction

High mortality and selective removal of fish caused by fishing can induce changes in the phenotypic composition of fish stocks (Enberg *et al.*, 2012; Heino *et al.*, 2015). Selective removal of specific phenotypes can erode overall trait diversity, and if traits under selection have a heritable component, evolutionary change can occur in a process known as fisheries induced evolution (FIE) (Richard, 2007; Redpath *et al.*, 2010; Heino *et al.*, 2013; Heino *et al.*, 2015). While the impacts and mechanisms of FIE are most frequently considered in a life-history context, specific behavioural and physiological traits can also render fish more susceptible to capture and may themselves be under selection in exploited fish stocks (Enberg *et al.*, 2012; Arlinghaus *et al.*, 2017; Diaz Pauli and Sih, 2017; Hollins *et al.*, 2018). Physiological and behavioural traits have been shown to correlate with susceptibility to capture in laboratory and field studies (Redpath *et al.*, 2010; Diaz Pauli *et al.*, 2015; Killen *et al.*, 2015a; Monk and Arlinghaus, 2017; Koeck *et al.*, 2018). However, these relationships can be inconsistent (Biro and Post, 2008; Redpath *et al.*, 2010; Wilson *et al.*, 2011; Vainikka *et al.*, 2016; Louison *et al.*, 2018), suggesting variation among species and/or context-dependency of relationships between phenotype and susceptibility to capture by fishing gears.

Understanding mechanistic links between individual traits and susceptibility to capture is essential for predicting the capacity of fishing gears to elicit phenotypic change in exploited populations (Horodysky *et al.*, 2015; Ward *et al.*, 2016; Hollins *et al.*, 2018) and provide effective fisheries management (Laugen *et al.*, 2014). Active gears (including trawls and seine nets) pursue or encircle targeted fish with nets, and exploit fish behavioural responses, including shoaling, to elicit capture. Physiological traits, such as individual swimming ability, may in this instance be linked to gear avoidance (Kim and Wardle, 2003; Killen *et al.*, 2015a), which in turn may be related to traits determining individual aerobic and anaerobic metabolic capacity. Specifically, aerobic scope [AS; the difference between an animal’s minimum (standard metabolic rate, SMR) and maximum rate of aerobic metabolism (maximum metabolic rate, MMR)] represents an individual’s capacity to supply oxygen to physiological processes beyond those required for maintenance, including aerobically powered ‘steady state’ swimming and recovery from exhaustive exercise. Additionally, the capacity to perform anaerobic metabolism may also be important for evading capture from active gear types as it represents an individual’s ability to perform rapid bouts of burst swimming, often employed in escaping predators, or performing other high-speed manoeuvres (Marras *et al.*, 2010).

Fish pursued by a trawl net may escape capture by sustaining high swim speeds or by exploiting potential escape routes to remove themselves from the path of pursuing gear. Individual fish with greater aerobic or anaerobic capacity may be more able to manoeuvre outside the path of active gear or endure the pace of a pursuing trawl net without succumbing to fatigue and falling back into the net (Winger, 2010; Killen *et al.*, 2015a; Hollins *et al.*, 2018) and thus maximising available opportunities for escape. Active gears may therefore selectively remove slower swimming fish or those with a lower capacity for aerobic or anaerobic metabolism from exploited populations. Individual variation in the behavioural response to fishing gears has also been observed, (Rose, 1995; Yanase *et al.*, 2009; Underwood *et al.*, 2015), with certain fish using escape routes to avoid an approaching trawl net (Brown and Warburton, 1999; Winger, 2010; Diaz Pauli *et al.*, 2015). The use of escape routes, rather than swimming to the point of fatigue in front of a trawl net, could modulate the relationship between metabolism and susceptibility to capture, however, the extent to which this occurs is currently unknown. It is also unknown whether the tendency to utilize escape routes is a repeatable characteristic of individual fish and whether social information may influence fish behaviour and encourage individuals to remain cohesive with conspecifics as the group swims ahead of the trawl net, falls back toward the oncoming net or as localized subgroups of individuals swim toward escape routes.

Behavioural responses of fish to active gears are influenced by extrinsic factors, including social interactions among the fish themselves (Rose 1995; Winger, 2010). Conformity of behaviour within a fish shoal can occur where members respond to stimuli, and these responses subsequently propagate through the shoal, manifesting as a collective behaviour (Brown and Laland, 2002; Magnhagen and Staffan, 2004; Marras *et al.*, 2011). While behavioural conformity may enable groups of fish to efficiently exploit escape routes in pursuing gears (Brown and Warburton, 1999) through a ‘follow the leader’ type principle, there is also evidence that it may increase susceptibility to capture of shoaling fish when the collective decision of a shoal is to voluntarily enter fishing gear, as opposed to escaping it (Rose, 1995; Thambithurai *et al.*, 2018). Trawls often exploit this behaviour to increase capture efficiency (Ryer, 2008) with gear components specifically designed to concentrate pursued fish ahead of a trawl mouth in a behaviour known as herding (Ryer *et al.*, 2009). Herding increases the density of fish in pre-existing shoals, and of shoals created by aggregating fish encountered individually or in smaller groups (Winger *et al.*, 2010). High densities of fish may exacerbate the role of social interactions in determining fish behaviour, and lead to shoals of fish comprised of individuals not normally associated with one another and/or are unfamiliar to each other. Shoals comprised of individuals unfamiliar with each other may be less cohesive (i.e. show greater between-individual distances) and therefore less likely to exhibit coordinated group-level responses (Chivers *et al.*, 1995; Atton *et al.*, 2014) or learn potential escape routes from conspecifics (Swaney *et al.*, 2001; Brown and Laland, 2002). This could generate a mechanism whereby the relationship between an individual’s phenotype and susceptibility to capture is modulated by the degree of familiarity with shoal mates. For example, a fish surrounded by unfamiliar individuals may be less influenced by its neighbours and so its vulnerability to capture may be determined by its own intrinsic traits, such as swimming ability.

By replicating the final moments of the capture sequence in a trawl fishery, using shoals of wild minnow (*Phoxinus phoxinus*), we investigate the role of individual metabolic traits in determining susceptibility to capture among shoals of either familiar or unfamiliar conspecifics. Trawling simulations were conducted within a laboratory swim tunnel, where fish were forced to swim ahead of a miniature trawl net. The degree of familiarity among shoal mates could influence the relationship between individual metabolic traits and vulnerability to capture in several ways. In shoals comprised of unfamiliar individuals, low shoal cohesion and decreased conformity of swimming behaviour may emphasize the importance of individual traits in determining vulnerability to capture. Alternatively, among familiar shoals, consistent leader/follow dynamics may lead to more repeatable behaviour around the trawl and so strengthen relationships between individual traits and vulnerability to capture.

## Materials and methods

### Experimental animals

All minnows *P. phoxinus* used in the study were sourced from the wild and collected using dip nets from the River Kelvin, UK. From the individuals collected, a total of 40 minnows were subsequently split across 10 aerated tanks (50 × 40 × 40 cm) so that each tank housed four individuals. All fish were acclimated to the laboratory for 7 months prior to experiments. Tanks were supplied with recirculating, UV-treated water maintained at 14°C. Each tank contained a shelter, gravel substrate and plastic plants, and all fish were kept on a 12L: 12D photoperiod. All tanks were shielded with opaque plastic blinds, preventing fish from interacting and observing individuals in neighbouring tanks. During this time, minnows were fed *ad libitum* a combination of commercial feed and bloodworm. Three months prior to the start of trawl trials and respirometry measurements, individual fish were sedated using benzocaine and given a unique combination of coloured VIE elastomer (Northwest Marine Technology Inc) to allow individual identification during trawl trials. We used common minnow (*P. phoxinus*) as the study species because they are a small shoaling fish (Magurran and Girling, 1986; Pitcher *et al.*, 1986) which lives in close association with the substrate, making this species a suitable representation of commercially targeted bentho-pelagic species.

### Familiar and unfamiliar trawl trials

Fish were subjected to a total of six trawl trials in groups of four fish: three trials with familiar fish and three trials with unfamiliar fish. Individual fish were haphazardly assigned to different ‘unfamiliar shoals’ before the beginning of the experiment and were assembled from fish in different tanks shortly before the start of each unfamiliar trial. Familiar shoals were assembled from fish housed in the same tank. Half of all fish encountered the trawl net in a ‘familiar’ context first, while the other half first encountered the trawl net in an ‘unfamiliar’ context. In all cases, fish within each shoal had encountered the trawl net an equal number of times as shoal mates and so within each shoal individual fish had the same degree of experience to trawling.

Trawling simulations followed a similar design to that of Killen et al. (2015a) and were conducted in a 90 l Steffensen-type swim tunnel (Loligo systems, Tjele, Denmark), designed to exercise fish at controlled speeds in laminar flow with a uniform velocity profile, thermoregulated to 14 ± 0.1°C. The working section of the swim tunnel was 70 long × 20 deep × 20 wide cm. A modified lid with a slit cut 30 cm from the front of the swim tunnel was used to allow for a perforated plastic divider to be placed between the fish and trawl net. This allowed the net to remain hidden from test fish during their settling period (see below) prior to the trial. The swim tunnel was calibrated using a vane wheel flow meter (Flowtherm NT, Höntzch, Waiblingen, Germany), with both the divider present and absent and with the trawl net in a deployed position to account for the added drag of the net on water velocity. Lastly, the setup was shielded from view by an opaque plastic blind to further minimize fish disturbance.

**Figure 1:**
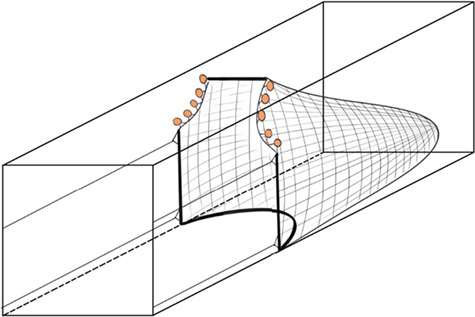
Layout of the miniature trawl net in the working section of the swim tunnel. Thick black lines indicate portions of the net that are flush with the walls of the swim tunnel, while portions of the net mouth bordered with small orange buoys indicate available escape routes.

Trawl trials consisted of introducing shoals of four individuals to the swim tunnel with the divider in place, preventing fish from interacting with the trawl prior to the start of the trial. With the lid firmly in place, water flow was increased to a speed of ~0.5 body lengths (BLs) per second (~2.5 cm s^−1^), based on the mean standard length of the minnows used. Fish were allowed to settle in the swim tunnel for 30 min. Following the settling period, water velocity was gradually increased from ~2.5 cm s^−1^ to ~66.5 cm s^−1^ over a period of 30 s, while the divider between the fish and trawl net was raised and the trawl net deployed. A custom-made scaled replica trawl net (Marine Institute, Memorial University of Newfoundland, Fig. 1) was used to simulate the final stages of capture in a trawl. Steel wire was threaded through the mesh along the mouth of the net to help maintain its shape and to ensure the sides of the net remained flush with the walls of the swim tunnel during trials. Two possible escape routes for fish were left in the top right and left corners of the swim tunnel (Fig. 1), each equal to ~6 cm^2^ or a third of the total cross-sectional area of the swim tunnel. Fish could swim freely back and forth through these routes for the duration of the trial. However, fish behind the net were still subjected to the oncoming flow of the swim tunnel but could partially avoid it by positioning themselves behind the footrope of the trawl. The bottom of the trawl net included rubber washers to simulate the rollers present on the footrope and provide weight to prevent the net from lifting during the trial. The top of the net included several orange beads spaced ~2 cm apart to replicate the appearance of floats along the headrope in a commercial trawl and provide a potential visual Cue for fish to orient near the front of the net. The trawl net was deployed externally behind the swimming shoal of fish by pulling a PVC handle attached to the trawl net by fishing wire. Once the net was 20 cm from the front of the swim tunnel, it was secured to prevent the net from slipping backwards during the trial.

**Table 1:** Different behaviours exhibited by fish in the trawl trials

**Behaviour/Event**	**Description**
Escape	Whole fish body passes behind net via an escape route, and remains behind the net, below escape route for 3 s
Re-Entry	Whole fish body re-enters the swim tunnel anterior to the trawl mouth via an escape route
In Net	Fish snout drops behind the mouth of the trawl
Out of Net	Fish tail moves ahead of trawl mouth

Where an escape occurred, the timestamp was taken from the beginning of the 3 s period.

Once the trawl net was deployed, fish were left to swim for 10 min. Swimming activity ahead of and within the trawl net, along with use of escape routes was recorded using high-definition video cameras (GoPro Hero 4 16:9 Full HD, 720p; GoPro, San Mateo, California, USA) mounted directly above and to the side of the swim tunnel. Fish were allowed a minimum 42 h recovery period between trials with no individuals used on two consecutive days. No adverse behaviours or fin damage was observed following the trawling trials and all fish resumed feeding once returned to their respective holding tanks.

### Analysis of fish behaviour around the trawl

To investigate the role of individual traits in determining time spent in the trawl net (a proxy of fishing vulnerability) and the degree of coordination among shoal members, video recordings of trials for each individual fish within each shoal were analysed by recording the cumulative time individual fish spent inside the net, as well as the number of successful escapes around the net (Table 1).

**Table 2:** Results of linear mixed effects models examining the role of metabolic traits and shoal composition on time spent in the net by individual fish

**Term**	**Estimate**	***SE***	***df***	***T***	***p***	***r***_***m***_^***2***^	***r***_***c***_^***2***^
Intercept	6.258	6.242	45.412	1.003	0.321	0.041	0.450
log (Mass)	−0.362	1.405	34	−0.258	0.798		
log (SMR)	1.558	2.352	46.524	0.662	0.511		
log (AS)	6.846	8.090	50.032	0.846	0.401		
log (MMR)	−8.024	10.200	50.117	−0.787	0.435		
log (EPOC)	−0.046	0.745	50.751	−0.061	0.951		
Shoal Composition	−2.700	4.691	169.446	−0.576	0.566		
log (SMR)^*^Shoal Composition	−0.554	1.834	169.446	−0.302	0.763		
log (AS)^*^Shoal Composition	−2.730	6.956	169.447	−0.392	0.695		
log (MMR)^*^Shoal Composition	4.297	8.793	169.447	0.489	0.626		
log (EPOC)^*^Shoal Composition	−1.297	0.641	169.448	−2.025	0.045		

### Measurement of metabolic traits

Once trawling trials were complete, all fish were subjected to intermittent flow respirometry after a 24 h fasting period to provide estimates of metabolic phenotype (SMR, MMR, AS and EPOC). Respirometry was undertaken at least 7 days after the cessation of trawl trials, with all respirometry completed within 12 days of the final trawl trial. Shoals of four individuals were removed from their holding tanks using dip nets, and two shoals (8 individuals total) were tested through respirometry concurrently. Estimates of MMR were achieved by manually chasing individual fish to exhaustion in a circular tank (50 cm diameter). Exhaustion was determined as the point at which fish were no longer receptive to the chase stimulus (mean chase time = 96 s, ± 34 s). The use of manual chase is assumed to elicit maximum rates of oxygen uptake as fish recover from prolonged anaerobic exercise (Killen *et al.*, 2017b). Once exhausted, fish were quickly transferred to individual cylindrical glass respirometry chambers (75 ml volume) attached to an intermittent flow respirometry system. Oxygen content of water within the closed respirometry circuit was recorded every 2 s using a firesting 4-channel oxygen meter and associated sensors (PyroScience GmbH, Aschen, Germany). The circuit itself comprised of a glass cylinder, and a length of gas impermeable tubing, through which water was constantly recirculated using a peristaltic pump. Respirometry chambers were submerged in an air saturated, temperature-regulated water bath (14°C ± 0.1°C, 50 l) and were shielded from disturbance and direct light via an opaque plastic blind. Every 8 min, an automated flush pump was programmed to turn on for 3 min and flush chambers with oxygenated water, and then switch off, sealing the respirometers to allow decreases in oxygen content due to fish respiration to be measured. Estimates of MMR were obtained by calculating rates of oxygen uptake for each 3 min time interval throughout the first 30 min of recovery immediately following exhaustive exercise. MMR (mg O_2_ h^−1^) was taken as the highest rate of aerobic metabolism during this period. After measurement of MMR, fish remained in their respective respirometry chambers overnight to allow for estimation of SMR and RMR. Individuals were removed from their chambers the following morning at around 09:00, having remained in the respirometers for ~17 h total. Once removed from respirometry chambers, fish mass and standard length were measured. Whole animal SMR (mg O_2_ h^−1^) was estimated as the lowest 10th percentile of measurements taken throughout the measurement period, while RMR was measured as the mean level of oxygen uptake, excluding the recovery period used for MMR estimation and the 4.5 h thereafter (Svendsen *et al.*, 2015). Excess post-exercise oxygen consumption (EPOC) for each fish was calculated from the area under the exponential recovery function, above RMR, until the time at which fitted values were equal to RMR. EPOC represents the increase in oxygen consumption above routine levels which occurs during recovery from a bout of exhaustive anaerobic exercise and is proportional to the anaerobic capacity of an animal (Lee *et al.*, 2003; Killen *et al.*, 2015a). Absolute AS was calculated as the difference between MMR and SMR.

### Statistical analyses

All statistical analyses were performed in R.3.5.1 (R Development Core Team) using the lme4 (Bates et al., 2015), MuMIn (Barton, 2015) and rptR (Stoffel *et al.*, 2017) packages. Time in net (T) was log (T + 1) transformed and used as the response variable for both repeatability estimates (hereafter *T_i_*) and statistical models investigating individual fish’s time in the trawl net. The effect of shoal composition (familiar versus unfamiliar) on time spent in the net was investigated using a linear mixed effect model (LME) via the function lmer, with log transformed time in net as the response variable, and log mass, log SMR, log AS, log MMR, log EPOC and shoal composition (categorical variable with two levels: familiar and unfamiliar) as explanatory variables. All possible interactions between individual metabolic traits and shoal composition were also included, with trial number (1–6) and Fish ID nested within tank as random effects. Models of best fit were determined using maximum likelihood estimation, although wherever a metabolic trait (SMR, AS, MMR, EPOC) remained in the model, mass was also retained to account for allometric scaling of metabolic rates regardless of AIC. Non-significant interactions were dropped sequentially, starting with those with the smallest t values, but were retained if their removal resulted in higher AIC values [ΔAIC > 2 (Arnold, 2010)]. Assumptions of homoscedasticity and normality of residuals were examined by visual inspection of residual plots. Significance testing, alongside model r^2^ values were used to indicate the strength of observed patterns. R^2^ values included marginal (r_m_^2^) and conditional (r_c_^2^) r^2^ values which indicate the variance explained by fixed factors, and by both fixed and random factors, respectively (Nakagawa and Schielzeth, 2013). Across-context repeatability of *T_i_* was calculated as adjusted repeatability, as described by Stoffel et al., (2017) using variances calculated with LMEs and including fish ID as a random effect. Repeatabilities of *T_i_* were also calculated at the shoal level for familiar and unfamiliar shoals as a measure of similarity of performance among fish within a given shoal.

To investigate whether fish behaviours around the trawl were synchronized within trials and between different shoal compositions, coefficients of dispersion (CD) for each behaviour were calculated for each 10 min trial (Chapman and Chapman, 1994; Hastie *et al.*, 2003; Killen *et al.*, 2018). Trials were split into 5 s time intervals, and the mean number of each type of event occurring within that interval recorded, alongside the variance around that mean. CD was then calculated as the variance/mean ratio across intervals, with values greater than 1 indicating temporally clustered events, and values less than 1 indicating events uniformly distributed in time.

## Results

From 240 observations of individual fish behaviour in groups around the trawl, a total of 138 entries into the net were observed. Of these, 65 were within the familiar treatment, and 73 within the unfamiliar treatment, with fish spending a mean time of 92 (± 151) and 70 (± 130) seconds in the trawl net, respectively. These means were not statistically different (t = −1.029, *P* = 0.3) and there was a great degree of variation among individuals within each treatment. The number of total recorded escapes was also similar between treatments, with a total of 162 escape events recorded in familiar trials and 177 escapes recorded in the unfamiliar trials.

**Figure 2:**
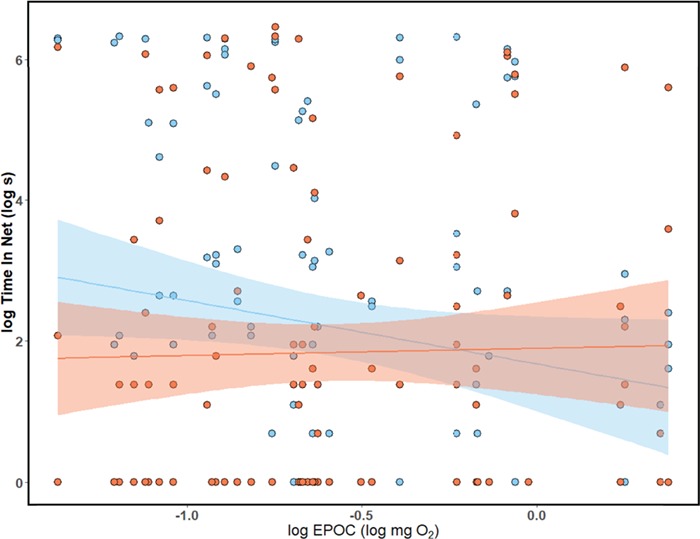
Relationship between anaerobic metabolic capacity (EPOC) and time spent in the net in familiar (blue) and unfamiliar (orange) shoals. Lines represent linear regression between log (EPOC) and log (time in net), for each shoal composition, while the shaded area corresponds to 95% confidence intervals. Three replicates are shown for each fish (*n* = 40) in both familiar (blue) and unfamiliar treatments (orange).

Adjusted repeatability for individual time in the trawl net was significant (R = 0.303, 95% CI: 0.16–0.44 *P* < 0.001), as were unadjusted repeatabilities calculated within each treatment (R = 0.26, 95% CI:0.01–0.4, *P* = 0.004 and R = 0.23, 95% CI:0.06–0.45, *P* = 0.01, for familiar and unfamiliar shoals, respectively). Repeatability of *T_i_* calculated within each trial (i.e. indicating similarity between the time spent in the trawl by a fish as compared to its shoal mates) were comparatively low and similar between each treatment (R = 0.13, 95% CI: 0–0.33, *P* = 0.06 and R = 0.03, 95% CI: 0–0.18, *P* = 0.4 for familiar and unfamiliar shoals, respectively). There was a significant interaction between log (EPOC) and shoal composition (*P* = 0.045), with log (EPOC) showing a weak negative association with time spent in the net when in a familiar shoal, but no relationship with time spent in the net when in an unfamiliar shoal (Table 2; Fig. 2) There were no relationships between any other individual-level traits and time in the net in either familiar or unfamiliar trials (Table 2; Fig. 2)*.*

**Figure 3:**
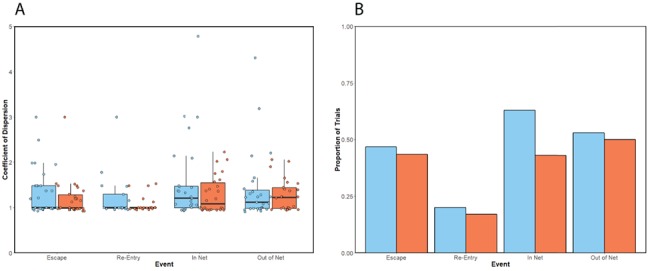
Temporal synchronisation of the four behaviours within a 5 s window (escapes, re-entries, in net, out of net) among four fish in familiar (blue) and unfamiliar (orange) trawl trials, as shown by CD ratios. Top: boxplot of CD for each behaviour. Each overlaid dot represents the CD calculated for one trial. Values > 1 represent events clustered in time. Boxplot upper and lower hinges represent the 25th and 75th percentiles, respectively, while the horizontal line within the box represents the median. Length of the whiskers represents the range of data points between each hinge, and 1.5× the difference between 25th and 75th percentiles. Bottom: bar chart showing the proportions of trials where CD ratio for each behaviour was > 1 (ie the number of trials were clustering for that behaviour was identified).

Successive behaviours of the same type (escapes, re-entries, entering the net and leaving the net) but performed by different individuals were found to be temporally clustered (Fig. 3). Clustering of events was recorded in a higher proportion of familiar than unfamiliar trials in all event types. However, these differences in clustering were generally small, except for entries into the net. In this instance, 20% more clustered fall-backs into the net were observed in familiar than unfamiliar trials.

## Discussion

Establishing mechanistic links between individual phenotypic traits and vulnerability to capture is an important component of fisheries management (Laugen *et al.*, 2014; Horodysky *et al.*, 2015; Ward *et al.*, 2016). In our study, we found that fish with higher anaerobic capacity spent less time swimming within the trawl net, in accordance with previous experimental work (Killen *et al.*, 2015a). However, the relationship was context specific and only present when fish were tested alongside familiar shoal mates. Evidence of synchronisation in behaviour around the trawl net was present in both familiar and unfamiliar shoals, suggesting that individuals within shoals are likely using a form of information transfer among individuals to avoid capture, as has been observed in previous experiments using trawl simulations (Brown and Warburton, 1999) and novel-avoidance tasks (Brown and Laland, 2002). This result supports the notion that while active gears have the capacity to selectively remove the slowest swimming fish, and drive phenotypic change in exploited fish stocks, extrinsic social factors may play a role in modulating this effect (Hollins *et al.*, 2018; Thambithurai *et al.*, 2018).

A negative relationship between individual anaerobic capacity (EPOC) and time spent in the trawl net was found in fish tested with familiar shoal mates only. Anaerobically powered, burst swimming is employed by fish during escapes from natural predators (Plaut, 2001) and is often observed *in situ* in fish pursued by trawls (Rose, 1995; Ryer *et al.*, 2009; Bayse *et al.*, 2016). In the present study, fish actively swam against the oncoming flow of water, using intermittent bursts of anaerobic swimming amidst periods of aerobic steady state swimming (Killen *et al.*, 2015a; Svendsen *et al.*, 2015). As trials progressed, fish gradually dropped further back where they would either begin to fall into the trawl net, or rise and make use of escape routes. Within the trawl mouth, fish would often employ successive bursts to move ahead of the trawl net, however, given the excessively high swim speeds and energy required to out swim the trawl net, this proved to be unsustainable as fish often made several passes into the mouth of the net over the duration of the trial.

Differences in the synchronisation of behaviours between fish in familiar and unfamiliar shoals may help explain the absence of relationship between anaerobic capacity and susceptibility to capture among unfamiliar shoals. Fish entering the net showed greater evidence of synchronisation in familiar shoals. This is likely attributable to greater shoal cohesion among fish swimming in familiar shoals (Chivers *et al.*, 1995), which would also explain why time spent in the net was more similar among fish within familiar than unfamiliar shoals. When swimming in a familiar shoal, the presence of conspecifics swimming ahead of the trawl net may provide added motivation for fish to leave the net once they drop behind the mouth (Barber and Wright, 2001; Atton *et al.*, 2014). As the shoal drops back into the net, individual fish may be more likely to engage in energetically costly anaerobic burst swimming to maintain cohesion with familiar conspecifics than if the shoal were comprised of unfamiliar individuals. The capacity for individual fish within a familiar shoal to regain a position ahead of the trawl net mouth subsequently becomes limitied by individual anaerobic capacity. In an unfamilair shoal, the perceived benefits of maintaining shoal cohesion may be reduced (Atton *et al.*, 2014) and so individual fish may be less willing to incur the costly metabolic debt strenuous swimming generates (Killen *et al.*, 2015a).

However, if fish in unfamiliar shoals were less inclined to enagage in anaerobic swimming to maintain a minimal distance from conspecifics, it would be expected that they would spend more time in the net on average than those swimming among a familiar shoal, as they would drop back into the net before the onset of fatigue. In our study, the opposite trend was observed, with average time in net being 22 s longer in familiar shoals, although the large degree of variability within both treatments makes interpreation difficult. An alternative explanation for the lack of relationship between anaerobic capacity and time in the net in unfamiliar shoals may be that decreased cohesion or coordination among unfamiliar shoals may emphasise the importance of stochastic processes, such as position of the fish relative to the trawl, in determining time spent in the net. A less cohesive shoal will collectively occupy more space than a more cohesive one, increasing the likelihood of a given fish swimming in close proximity to a potential escape route. In this instance, a fatigued fish falling back toward the trawl net may have greater opportunity to access an escape route purely by chance, effectively missing the net. This presents a mechanism whereby a fatigued fish may completely avoid spending time in the net, weakening the relationship between metabolic traits and susceptibility to capture, and also decreasing synchronisation of fish successively entering the trawl mouth.

Fish pursued by a trawl net likely do so among an assortment of other species and a mixture of familiar and unfamiliar conspecifics (Rose, 1995; Ryer, 2008; Underwood *et al.*, 2015). In this experiment, we show that simply altering the composition of a shoal can influence gear selectivity and phenotypic drivers of fishing selection. However, the cues elicting herding in our experiment differ from those experienced by pursued fish in a trawl in the wild. In our experiment, the on-coming flow of the swim tunnel provides a strong cue for fish to orient themselves perpendicular to the trawl mouth and maintain forward swimming. In the wild, while herding cues provided by trawl doors and sediment plumes caused by the trawl sweeps’ contact with the seafloor encourage fish to aggregate together, (Winger, 2010), their orientation and swimming direction relative to different parts of the trawl can vary widely (Ryer, 2008; Yanase *et al.*, 2009; Winger, 2010; Underwood *et al.*, 2015). As a result, in the wild, fish may turn headfirst into a pursuing trawl, (Rose, 1995; Brown and Warburton, 1999; Underwood *et al.*, 2015), increasing capture and leading to the entry of fish not yet fatigued (Rose, 1995; Yanase *et al.*, 2009). Fish may also turn headfirst into pursuing trawls in an effort to maintain minimal distance from, or keep visual contact with shoalmates, also leading to the capture of non-exhausted fish (Day *et al.*, 2001). To study the effect of fish entering a net head-first, the use of remotely controlled artificial fish could be adopted in a laboratory setting (Faria *et al.*, 2010; Bierbach *et al.*, 2018) and would provide an interesting avenue for further research. The trawl used in this experiment was static and simulated the pursuit of fish using an on-coming flow of water. As such, this experiment does not replicate the hydrodynamic disturbances caused by a full-scale trawl as it moves through the water, which may provide additional cues for fish to exploit when avoiding capture. These effects could not be replicated in the experimental approach used here, and their effects on fish behaviour and capacity to escape a pursuing trawl warrant further research. In addition to aggregating fish unfamiliar with one another within a pursued shoal, trawls may also cause shoals with phenotypic compositions that are unusual [for example, a shoal largely comprised of asocial individuals, (Cote *et al.*, 2012)], or would normally not arise in natural settings. Specifically, investigating how varying the phenotypic composition of a shoal influences the vulnerability of its respective members to capture (Killen *et al.*, 2017a; Bevan *et al.*, 2018) would be a useful next step. While there was no evidence of fish learning to avoid trawl capture in this study, fish have shown the capacity to learn trawl avoidance (Pyanov, 1993; Özbilgin and Glass, 2004), and the numerous negative stimuli provided by a trawl (e.g. vessel noise, forced exhaustive swimming) may provide sufficient negative reinforcement for fish to learn escape behaviours in the wild. Investigating how the inclusion of fish exhibiting learned trawl escape behaviours influences shoal mates’ vulnerablity to capture and how this may influence selection on physiological traits therefore warrants further study.

Neither SMR, MMR or AS were related to vulnerability to capture in either familiar or unfamiliar shoals. Aerobically powered red muscle fibres contribute to fish swimming performance once the fish has begun to swim beyond its gait transition speed (McKenzie, 2011). While this contribution at high swim speeds is comparatively small, a fish with greater aerobic capacity may postpone the recruitment of anaerobic muscle fibres in its efforts to maintain station ahead of the trawl net, and thus delay its eventual fatigue resulting in decreased time in the net. In this experiment, AS had further capacity to influence vulnerability to capture, determining the available metabolic budget which could be allocated to the restoration of homeostasis after engaging in strenuous exercise (McKenzie, 2011; Killen *et al.*, 2015b). The fact that no relationship between susceptibility to capture and either AS or MMR was found, suggests that within familiar shoals anaerobic capacity was a more important influence in determining time fish spent in the net.

The results of this study support the notion that commercial harvest using active gears has the potential to alter the phenotypic composition of exploited fish stocks by selectively removing the poorest swimming fish (Killen *et al.*, 2015a; Hollins *et al.*, 2018). However, this experiment also shows that this effect is context specific, making the strength of this selectivity in the wild difficult to determine. The impact of this erosion of phenotypic diversity is difficult to predict, but evolutionary impacts of fisheries harvest has implications for the recovery and sustainability of exploited fish stocks (Enberg *et al.*, 2009; Heino *et al.*, 2013; Laugen *et al.*, 2014), as well as the economic viability of fisheries themselves (Eikeset *et al.*, 2013). Considering the role of anaerobic metabolism in predator prey dynamics (Kaufman *et al.*, 2006; Killen *et al.* 2015a), selective removal of slow swimming individuals could result in exploited fish stocks becoming less available to predators, or otherwise alter food web dynamics (Claireaux *et al.*, 2018). Fish exhibiting high exercise performance can also exhibit prolonged recovery times after engaging in exhaustive exercise experienced during predator avoidance or catch and release angling (Clark *et al.*, 2017), likely attributable to the excessive metabolic debt these high performance fish can incur. The high performance swimmers exhibiting high anaerobic metabolic capacity that are more likely to escape trawl capture may therefore also be those most likely to succumb to delayed mortality after exhaustive exercise (Clark *et al.*, 2017), particularly under future scenarios of climate warming (Brownscombe *et al.*, 2014; McLean *et al.*, 2016; Clark *et al.*, 2017). Selection for high performance phenotypes driven by active fisheries may therefore leave exploited stocks particularly maladapted to the effects of climate change, with further implications for catch and release fisheries targeting species which are also subject to harvest by active gears, such as Atlantic cod (*Gadus morhua*).

To summarize, vulnerability to trawling was linked to individual anaerobic capacity in fish swimming among familiar conspecifics, but this relationship was absent when fish faced the trawl alongside an unfamiliar shoal. This pattern seems to be driven by differences in shoal cohesion or coodination and subsequent collective behaviour of fish. While vulnerability to capture showed significant across-context repeatability, as well as a context-specific relationship with anaerobic capacity, both these trends were weaker than those observed in previous experimental work (Killen *et al.*, 2015a). This is likely attributable to the inclusion of escape routes around the trawl, allowing behavioural responses beyond keeping pace in front of the trawl to contribute to lowering individual time in the net. This study demonstrates a mechanism where relationships between a fish’s metabolic traits and vulnerability to capture in a trawl may be modulated by a individual’s social context. These results have implications for determining the potential strength of selection on individual fish traits by active gears and how these relationships may be modulated by extrinsic factors.
